# Umbilical venous access through the ductus arteriosus for arterial embolization of a neonatal sacrococcygeal teratoma

**DOI:** 10.1186/s42155-025-00634-y

**Published:** 2026-04-17

**Authors:** Vicente Belloch-Ripollés, Fernando Gómez-Muñoz, José Martínez-Rodrigo, Ali Boukhoubza, Javier Gómez-Chacón, Alfredo Marco-Macián

**Affiliations:** Hospital Universitario y Politécnico La Fe, Avenida Fernando Abril Martorell, Valencia, 106, 46026 Spain

**Keywords:** Sacrococcygeal teratoma, Embolization, Newborn, Ductus arteriosus, Umbilical vein access, Interventional radiology

## Abstract

**Background:**

Sacrococcygeal teratoma (SCT) is the most common germ cell tumor in children and the most frequent fetal neoplasm. The treatment of choice is complete surgical resection. Preoperative endovascular embolization has proven to be a safe and effective technique to reduce bleeding rates before and during surgery. In previously reported cases, the vascular access chosen for embolization was always arterial (carotid, subclavian, or femoral). However, in neonates, the small caliber of arteries, their greater propensity for spasm, and the risk of puncture-related complications sometimes make the vascular access the most challenging step of the procedure.

**Case presentation:**

In this work, the authors present a case of successful preoperative embolization of an SCT in a newborn on the first day of life, using an innovative vascular approach to control perioperative bleeding by accessing the arterial circulation through the ductus arteriosus via umbilical venous access. This technique allowed safe and effective embolization while avoiding arterial puncture.

**Conclusions:**

While anatomical feasibility depends on ductus arteriosus patency, this approach may be valuable in selected neonates with SCTs where femoral access is technically challenging. Additional advantages include avoiding arterial puncture, thus reducing the risk of arterial spasm, thrombosis, or hemorrhage, and the fact that the umbilical vein is an already commonly used access in pediatrics, thereby reducing the risks inherent to neonatal arterial access and achieving comparable technical success.

## Background

Sacrococcygeal teratoma (SCT) is the most frequent congenital germ cell tumor in neonates [1]. The standard of treatment consists of complete removal of the tumor, with early resection being associated with a better prognosis [2]. However, surgical resection can be complex, with a high risk of uncontrolled tumor bleeding and hemodynamic instability, which can be life-threatening for the newborn.

Lesions larger than 10 cm are associated with high perinatal mortality, especially if they are hypervascular [1]. High-output heart failure, internal tumor bleeding, and perioperative bleeding are the most common causes of neonatal morbidity and mortality [1]. These tumors are usually supplied by a hypertrophic median sacral artery, which may be similar in size to the common iliac artery and cause vascular steal syndrome. The tumor may also be supplied by both branches of the internal iliac artery, primarily through lateral sacral arteries [3].

Preoperative embolization can reduce intraoperative bleeding; nevertheless, arterial access in neonates carries technical challenges and potential complications. There are few cases described in the literature of preoperative endovascular embolization of SCT. In the cases described, vascular approaches through the left common carotid artery [1, 2, 4], left subclavian artery [3], or right common femoral artery [5, 6] have been used.

These studies demonstrate that preoperative endovascular embolization is safe and effective, reducing the rate of bleeding before and during surgery, making laparotomy unnecessary to ligate the feeding arteries.

We describe a case of successful neonatal SCT arterial embolization avoiding arterial puncture, using an umbilical venous access and crossing through the ductus arteriosus.

## Case presentation

**Fig. 1:**
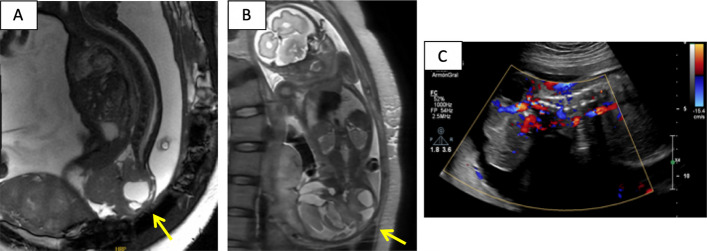
Fetal MRI (**A** and **B**) and transabdominal ultrasound Doppler study (**C**) at week 29 + 6. Solid-cystic sacrococcygeal tumor corresponding to a teratoma (type 2 of the Altman classification) (arrows in **A** and **B**), highly vascularized in the Doppler study (**C**)

A 44-year-old woman, pregnant via in vitro fertilization, was diagnosed with a type II SCT (Altman classification) at 21 + 5 weeks’ gestation after a routine 20-week ultrasound revealed a heterogeneous mass caudal to the sacrum. Fetal MRI confirmed a solid-cystic tumor. The patient decides to continue with the pregnancy and periodic clinical and radiological check-ups are performed during the pregnancy. Serial imaging at 29 + 6 weeks’ gestation demonstrated progressive growth and hypervascularization of the SCT (Fig. 1).

At 34 + 6 weeks, the patient developed severe polyhydramnios, shortened cervical length, and elevated middle cerebral artery peak systolic velocity, suggesting severe fetal anemia. Because of the suspicion of progressive fetal decompensation that could lead to heart failure and fetal hydrops, a multidisciplinary team (MDT) decided to terminate the pregnancy by cesarean section.

A female neonate weighing 2600 g (estimated 2300 g excluding the tumor) was delivered with Apgar scores 9/9. Given the tumor’s high vascular supply and large proportion of cardiac output, MDT decided preoperative embolization and was planned for day 1 of life to reduce bleeding risk and allow a few days of hemodynamic adaptation before surgery.

### Procedure

**Fig. 2:**
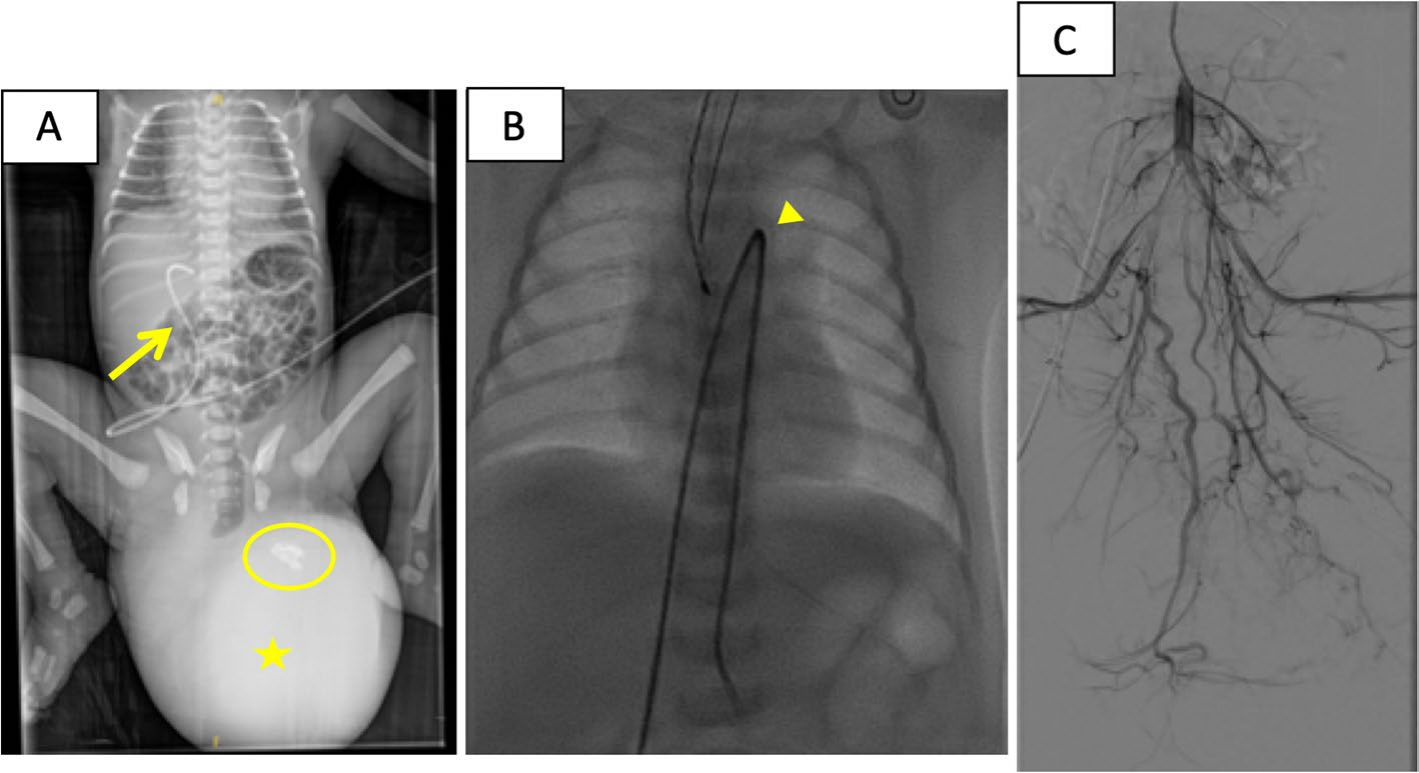
Abdominal radiograph on the first day of life (**A**) highlights a soft tissue mass caudal to the sacrum (star), with coarse calcification within it (circle), corresponding to the SCT. The patient has an umbilical vein already cannulated (arrow) by the pediatricians. Fluoroscopy image (**B**), of the catheter crossing through the ductus arteriosus (arrowhead) to the descendent aorta. Arteriography (**C**) from the aortoiliac bifurcation, identifying profuse vascularization of the SCT dependent on a hypertrophic middle sacral artery and branches of both internal iliac arteries

A 4-Fr introducer is placed in the already cannulated umbilical vein (Fig. 2A), exchanging it over a guidewire. Using a guidewire and Bern catheter, systemic arterial access to the aorta was obtained through the ductus arteriosus (Fig. 2B). Angiography from the aortoiliac bifurcation revealed profuse tumor vascularization from a hypertrophic median sacral artery and branches of both internal iliac arteries (Fig. 2C).

**Fig. 3:**
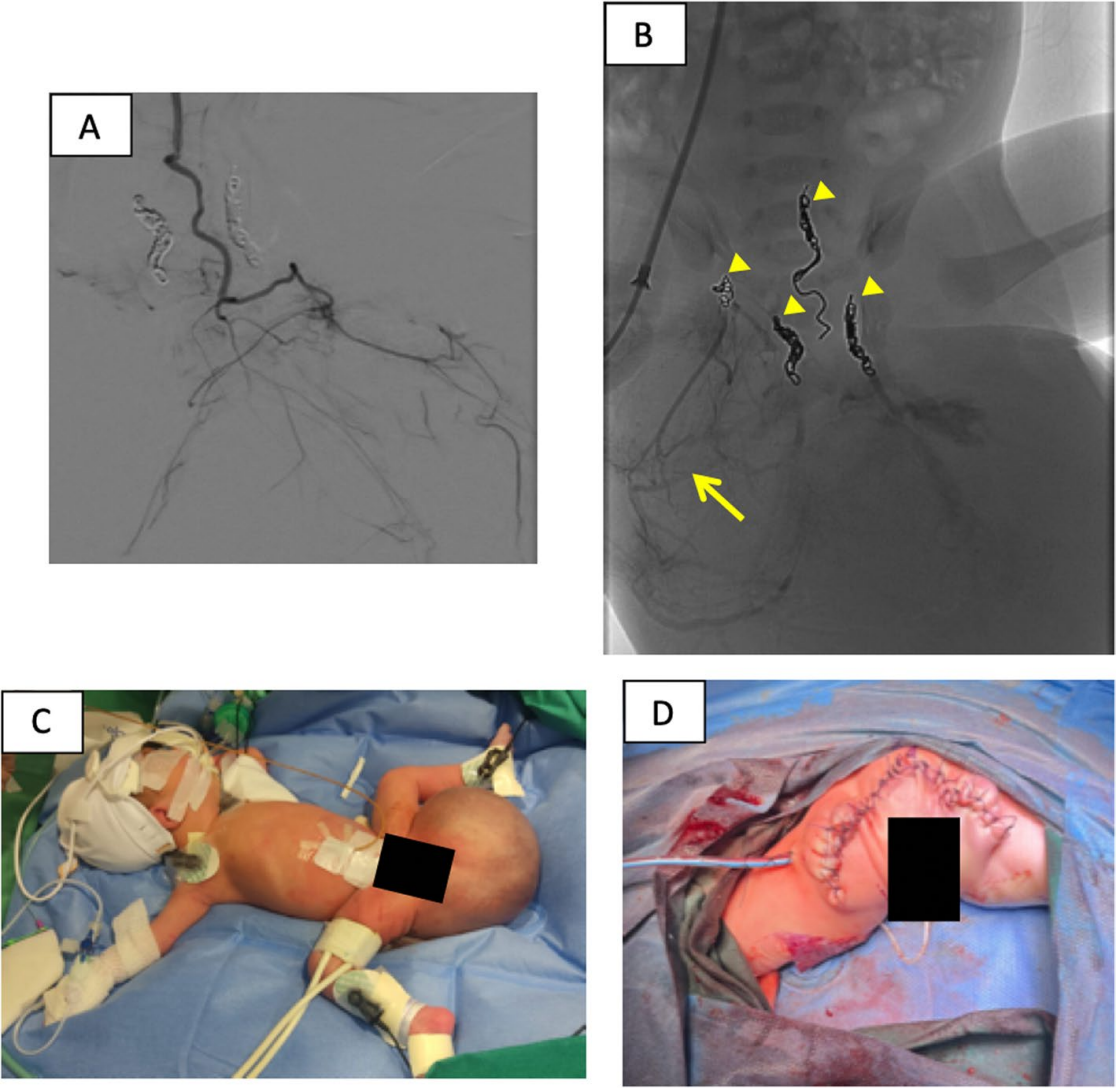
Selective arteriography (**A**) of a feeding artery of the SCT coming from a branch of the left internal iliac artery. Final fluoroscopic image (**B**) depicts contrast retention in the arteries embolized with Embocube (arrows) and coils (arrowheads). **C** Subtle changes in the skin color of the tumor immediately after embolization. **D** A prone image of postoperative perineal reconstruction

The different afferent arteries of the SCT (hypertrophic middle sacral artery and branches of both internal iliac arteries) are selectively catheterized and embolized with a 2.4-Fr Progreat microcatheter (Fig. 3A). Distal embolization was performed with Embocube (*Merit Medical*) diluted in contrast, followed by proximal coil occlusion (2–3 mm diameter of each coil) (Fig. 3B).

Total contrast usage during the procedure was 16 cc, 12 cc for angiographic series plus 4 cc mixed with the Embocube for embolization. The final angiogram confirmed complete devascularization. No complications occurred related to the access site, the technique, or the embolized area (Fig. 3C).

### Outcome

Elective surgical resection performed 3 days after arterial embolization was technically successful without significant blood loss (Fig. 3D). Pathology confirmed a mature solid-cystic SCT measuring 11.6 cm in maximum diameter. The neonate was discharged at 26 days of age, 25 days after embolization and 22 days after surgery, with adequate oral tolerance, normal stools, and spontaneous urination.

## Conclusions

No cases have been found in the literature in which an approach through the umbilical vein was used, taking advantage of one of the characteristics of neonatal circulation: the ductus arteriosus, which connects the pulmonary and systemic circulations. This case demonstrates a novel use of umbilical venous access for arterial embolization, avoiding arterial puncture, thanks to being able to pass into the systemic circulation through the ductus arteriosus.

Advantages of this approach include:

- Reduced risk of arterial spasm, thrombosis, or hemorrhage
- Use of a routinely placed neonatal access
- Facilitation of median sacral artery catheterization without supra-aortic puncture

The position of the legs due to the size of the tumor often prevents a femoral approach. Catheterization of the median sacral artery is more favorable from a supra-aortic approach, and passage to the aortic arch through the ductus arteriosus eliminates the need for carotid artery puncture for this purpose. The small size of the arterial access, its greater propensity for arterial spasm, complications secondary to arterial puncture, and the fact that the umbilical vein is a commonly used access in pediatrics are advantages of the described approach.

While anatomical feasibility depends on ductus arteriosus patency, this technique may be valuable in selected neonates with SCTs where femoral access is technically challenging.

In conclusion, the umbilical venous access through the ductus arteriosus is a safe and feasible route for preoperative arterial embolization of neonatal SCTs for controlling perioperative bleeding and reducing the risk of perinatal morbidity and mortality, avoiding arterial puncture and potentially reducing complications of the puncture site.

## Data Availability

All data generated or analyzed during this study are included in this published article, for further contact with the corresponding author.

## Abbreviations

SCT: Sacrococcygeal teratoma
MDT: Multidisciplinary team
